# How to objectively assess and observe maladaptive pain behaviors in clinical rehabilitation: a systematic search and review

**DOI:** 10.1186/s40945-021-00109-y

**Published:** 2021-06-03

**Authors:** Florian Naye, Chloé Cachinho, Annie-Pier Tremblay, Maude Saint-Germain Lavoie, Gabriel Lepage, Emma Larochelle, Lorijane Labrecque, Yannick Tousignant-Laflamme

**Affiliations:** 1 School of Rehabilitation, Faculty of Medicine and Health Sciences, Université de Sherbrooke, Sherbrooke, QC Canada; 2 62 Rue de la Rondonnerie, 45120 Corquilleroy, France; 3 Research Center of the Centre Hospitalier Universitaire de Sherbrooke, Sherbrooke, QC Canada

**Keywords:** Pain behavior, Assessment, Protective behavior, Endurance behavior, Avoidance behavior, Musculoskeletal pain

## Abstract

**Background:**

Cognitive-affective factors influence the perception of pain and disability. These factors can lead to pain behaviors (PB) that can persist and become maladaptive. These maladaptive PB will further increase the risk of chronicity or persistence of symptoms and disability. Thus, clinicians must be prepared to recognize maladaptive PB in a clinical context. To date, in the context of assessment in a rehabilitation setting, PB in clinical settings are poorly documented. The main objective of this study was to identify direct observation methods and critically appraise them in order to propose recommendations for practice. As a secondary objective, we explored and extracted the different observable PB that patients could exhibit and that clinicians could observe.

**Methods:**

We conducted a comprehensive review on four databases with a generic search strategy in order to obtain the largest range of PB. For the first objective, a two-step critical appraisal used clinical criteria (from qualitative studies on barriers to implement routine measures) and psychometric criteria (from Brink and Louw critical appraisal tool) to determine which observation methods could be recommended for clinical practice. For the second objective, we extracted PB found in the literature to list potential PB that patients could exhibit, and clinicians could observe.

**Results:**

From the 3362 retrieved studies, 47 met the inclusion criteria for the first objective. The clinical criteria allowed us to select three observation methods. After the psychometric step, two observation methods were retained and recommended for clinical practice: the Behavioral Avoidance Test-Back Pain (BAT-Back) and the Pain Behaviour Scale (PaBS). For the second objective, 107 studies met the inclusion criteria. The extraction of the PB allowed us to list a large range of PB and classify the data in 7 categories of PB.

**Conclusion:**

Our results allowed us to recommend two observation methods for clinical practice. However, these methods have limitations and are validated only in chronic low back pain populations. With the extraction of PB presented in the literature, we contribute to better prepare clinicians to recognize PB in all patients who are experiencing pain.

## Introduction

The biopsychosocial model of pain strongly supports that in addition to biological and social factors, cognitive-emotional factors drive the experience of pain and disability [1–4]. According to a systematic review of the best practice care for musculoskeletal pain, the authors conclude that the assessment of psychosocial factors should be an essential part of the evaluation process [5]. This suggests that the evaluation of maladaptive cognitions and emotions should specifically be assessed by rehabilitation professionals.

According to the Fear-Avoidance Model, maladaptive cognitions (e.g., Pain catastrophizing) and maladaptive emotions (e.g., Fear of movement) may contribute to the development of avoidance-related pain behaviors (PB) [6]. In addition to the avoidance patterns, the Endurance-Avoidance Model proposes that thought suppression or distraction may lead to endurance-related pain behaviors [7], namely, the opposite of the avoidance behaviors. The persistence of these PB may lead to poor outcomes and are known risk factors for the recurrence of pain and chronicity [8–10].

PB are defined as “the behavioral alterations observed in individuals experiencing pain” [11] and consist of two main categories. The first category includes protective PBs, which is defined as “any action primarily aimed at minimizing the experience of pain, promoting recovery from injury, or reducing the probability of further injury” [11] (e.g. avoiding a threatening task). The second category includes communicative PBs, which is defined as “observable behaviors meant to communicate to others that one is experiencing pain” [12] (e.g. touching the painful area after task performance). Some could argue that protective PB may also serve as a communicative function when they are viewed by others, and that communicative behaviors may also serve to seek support or assistance from the patient’s social environment [11]. By definition, these categories are only applicable to avoidance behaviors.

However, since the definition of PBs covers a large range of behaviors such as vocalizations, sighing, rubbing, posture modification, and movement modification, the interpretation of the PB as adaptive or maladaptive is often difficult. A specific PB may be adaptive in the short term (e.g. relative rest after injury), but may become maladaptive if it persists [9] or becomes more frequent [13]. A PB may have negative outcomes in the short term but may have a positive outcome in a long-term (e.g. a return to physical activity leads to an increase in pain in the short term, but a decrease in pain in the long term) [14, 15]. Moreover, the contextual and social environment can also modify the manifestations of the PB [16, 17].

As maladaptive pain behaviors can be expressed in many different ways [18], clinicians can often struggle to detect relevant findings in a clinical environment. Knowledge of maladaptive behaviors is critical in understanding, assessing, and treating persistent pain [19]. Yet, to our knowledge, there is no review documenting the observation methods to objectively assess PB in patients with musculoskeletal pain. The main objective of this study was to identify direct observation methods and critically appraise them in order to propose recommendations for practice. As a secondary objective, we explored and extracted the different observable PB that patients could exhibit and that clinicians could observe.

## Methods

### Design

We chose a *systematic search and review* to answer our main objective. This design combines the strengths of a critical review with a comprehensive search process that typically addresses broad questions to produce a synthesis of best evidence [20]. We aimed to answer this specific research question: What are the direct observation methods, adapted to clinical settings, to assess PBs in an adult population (≥18 years-old) experiencing musculoskeletal pain. For our second objective, we chose a narrative review to present the PBs identified from the literature.

This review was registered with the PROSPERO database: CRD42018093102.

### Identification and selection of studies

For both objectives, four (4) databases (CINAHL, PubMed, PsycInfo, Scopus) were explored. Literature addressing observable pain behavior was examined using the most generic search strategy: (“pain behavior” OR “pain behaviour” OR “avoidance behavior” OR “endurance behavior” OR “avoidance behaviour” OR “endurance behaviour”) NOT (animal OR animals OR mice OR mouse OR rat OR rats OR dog OR dogs OR rodent OR rodents OR murine OR adolescent OR adolescents OR child OR children OR pediatr* OR “cognitive impaired” OR “cognitive impairment”) with title filter. The choice of a generic search strategy was based on the intention to target the largest range of studies on pain behavior. Also, we used title filter to focus on the literature that the purpose is specific to PB. Only literature published in English and French was included. This search was performed in March 2020 thus the search period was from inception to March 2020.

After removing duplicates, the screening of the records was made by two independent evaluators (CC, and FN) who screened the study titles and abstracts to identify eligible articles for the full-text review. For this first step, the selection was based on common criteria for the two objectives. To be included, the potential studies had to present content meeting three inclusion criteria. Because of the abundance of literature on the specific topics of pain in people with cognitive and communicative impairments, we decided to add two exclusion criteria (detailed inclusion/exclusion criteria are presented in Table 1).

**Table 1:** Selection criteria

RECORDS SCREENING	
For the two objectives	
Inclusion criteria	1) Observable behaviors related to the experience of pain 2) Human participants 3) Adult participants (> 18 years-old)
Exclusion criteria	1) Participants with cognitive or communicative impairments 2) Studies in other language than English and French
FULL-TEXT ASSESSMENT FOR ELIGIBILITY	
For the first objective: *Observation methods to assess pain behaviors*	
Inclusion criteria	1) Participants with musculoskeletal pain 2) Use of a direct observation method with enough details to reproduce it
For the second objective: *Pain behaviors present in the selected literature*	
Inclusion criterion	1) behavior that can be directly observed by clinicians

The assessment for eligibility of the full-text articles was made by the same two independent evaluators (CC, and FN). This assessment presented specific criteria for each objective. For the first objective, two inclusion criteria were applied (Table 1). For the second objective, one inclusion criterion was applied (Table 1). Because pain is not specific to a condition, we broadened the selection of studies to include all populations for objective 2. The final decision on article inclusion was made by consensus. In case of disagreement, a third reviewer (YTL) was available to make the final decision.

### Critical appraisal of assessment tools (1st objective)

Because one of the aims of a systematic search and review is to make recommendations for practice, we developed a triage process to further refine the selection before extracting the data. The triage process was based on clinical and psychometric aspects. We used the reported barriers to implement outcome measures from qualitative data to determine relevant clinical criteria [21, 22]. To be included, the tool had to meet each of these clinical criteria:

- The time to complete the observation method had to be equal or less than 10 min. In case of observation during a more comprehensive assessment (clinicians obtain more information than PB alone), this procedure had to be equal or less than 30 min.
- The scoring method had to be made without the use of videotaping.
- An interpretation of the score to help clinicians in their care plan had to be inherent to the tool.
- The tasks performed during the observation method had to require no special equipment and had to be made with commonly-used equipment (if required).

Afterwards, the studies that met the clinical criteria were methodologically appraised for their measurement (psychometric) properties based on the Critical Appraisal Tool (CAT) developed by Brink and Louw [23]. The CAT consists of a 13-item checklist to assess the validity and reliability of clinical instruments. We removed items three, seven, nine, and eleven as they were specific to concurrent validity and not relevant to the nature of our analysis. As other items were conditional, some items could be rated as not applicable. To estimate the study quality (based on the CAT), we used the ratio (percentage) between the number of items with a positive answer (yes) and the total number of relevant items [24]. We used a cut-off of 60%, where a given tool was rated > 60%, it was deemed acceptable and retained for further analysis [25]. All appraisal-related procedures were made by two independent evaluators (CC, FN). In case of disagreement, a third evaluator (YTL) was available to make the final decision.

### Data extraction and data analysis

Two independent evaluators (CC, FN) extracted the data from the retained observation methods. A third evaluator (YTL) verified the extraction.

For the first objective, we extracted the data regarding: the aim of the observation method, its clinical administration, the observed PB, the scoring and its interpretation, the clinical benefits of the method, the result of the statistical analysis of validity and reliability, and the target population. A narrative synthesis was made to inform clinicians about the characteristics of each recommended observation method. For the second objective, all observable PB were extracted and regrouped into homogenous categories.

## Results

### Selection of the studies

**Fig. 1:**
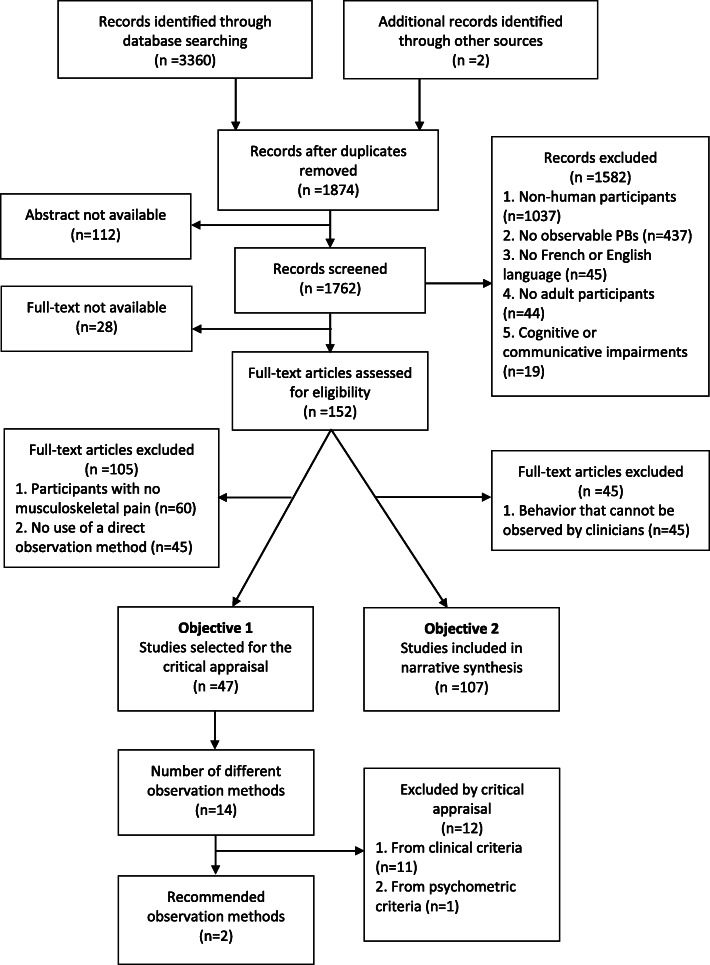
Flow chart

For the first objective, 3360 relevant articles were found in the various databases consulted. Two more articles were included after an exploratory hand search. After the removal of duplicates (1488 excluded), title/abstract screening (1694 excluded with 112 abstracts not available), we obtained a pool of 180 articles. From this pool, 28 articles were not available and, 105 articles failed to meet our inclusion parameters, which left 47 articles for dedicated assessment (see Fig. 1 for the flow chart diagram).

### Critical appraisal

From the 47 articles, we found 14 different observation methods. From the 14 initial methods, 9 used videotaped sequences to determine the number of different PBs during task execution which considerably limit their use in the clinic setting. Also, 11 observation methods did not propose an interpretation of the score which made it difficult to use these data to determine or adapt the treatment plan. Table 2 presents the extracted data for the assessment of the clinical criteria. Only three observation methods met all clinical criteria: 1) the Behavioral Avoidance Test-Back Pain (BAT-Back) [27], 2) the Pain Behavior Scale (PaBS) [36], and 3) the Test Instrument for Profile of Physical Ability (TIPPA) [37].

**Table 2:** First step of the critical appraise: clinical criteria appraisal

Method	Time to complete ≤ 10 min OR ≤ 30 min if part of a comprehensive assessment	Scoring method Not videotaped	Interpretation Does the method propose an interpretation of the score (severity, …)?	No special equipment required	Decision (retain or reject)
Aung’s method [26]	We can assume < 10 min				Reject
Behavioral Avoidance Test-Back Pain (BAT-Back) [27]	< 10 min	Specific rating according to the level of avoidance	Range of possible scores is 0 to 60 (0 = no avoidance, 60 = every movement is avoided)		**Retain**
Butler and Kozey’s method [28]	We can assume < 10 min				Reject
Cinciripini’s method [29]	We can assume < 10 min				Reject
Cold pressure method [30]	2-min				Reject
Keefe and Block [31] (K&B) and modified K&B [32]	10-min				Reject
Keefe’s walk method [33]	We can assume < 10 min				Reject
Koho’s method [34]	We can assume > 10 min				Reject
Moores’ method [35]	We can assume > 10 min	Frequencies of PB			Reject
Pain Behaviour Scale (PaBS) [36]	Part of a physical performance test of 10 to 15 min	Frequencies of PB	A total score of severity can be determined from 0 to 15		**Retain**
Prkachin’s method [18]		Number of PB			Reject
Test Instrument for Profile of Physical Ability (TIPPA) [37]	Part of a comprehensive assessment but we can assume < 30 min	Presence of PB	A severity scale is proposed based on the number of activities with PBs		**Retain**
Thieme’s method [38]	8-min				Reject
Watson’s method [39]	We can assume > 10 min				Reject

: unclear

: no

: yes

The methodology of the three selected observation methods was then evaluated using the CAT. The CAT was modified for the BAT-Back and the TIPPA, as these two methods only analyzed the inter-rater reliability. Items 5 (Raters blindness in intra-rater reliability), 6 (Variation of the order of examination), and 8 (Stability of variable) were not applicable. The BAT-Back obtained a percentage of 66.7%, the PaBS obtained a percentage of 77.8%, and the TIPPA obtained a percentage of 16.7%. Given the score below the a priori threshold of 60%, the TIPPA was not retained for further analysis. Table 3 presents the completed CAT scores for the 3 instruments.

**Table 3:** Second step of the critical appraisal: psychometric criteria according to the Critical Appraisal Tool [23]

	Item from the CAT	BAT-Back	PaBS	TIPPA
1	If human subjects were used, did the authors give a detailed description of the sample of subjects used to perform the (index) test?	Yes	Yes	No
2	Did the authors clarify the qualification, or competence of the rater(s) who performed the (index) test?	No	Yes	No
4	If interrater reliability was tested, were raters blinded to the findings of other raters?	No	Yes	No
5	If intrarater reliability was tested, were raters blinded to their own prior findings of the test under evaluation?	n/a	Yes	n/a
6	Was the order of examination varied?	n/a	No	n/a
8	Was the stability (or theoretical stability) of the variable being measured taken into account when determining the suitability of the time interval between repeated measures?	n/a	No	n/a
10	Was the execution of the (index) test described in sufficient detail to permit replication of the test?	Yes	Yes	No
12	Were withdrawals from the study explained?	Yes	Yes	Yes
13	Were the statistical methods appropriate for the purpose of the study?	Yes	Yes	No
	Ratio between the number of items with a positive answer and the total number of “applicable” items	4/6	7/9	1/6
	Percentage	66.7%	77.8%	16.7%
	**Decision before extraction**	***Retain***	***Retain***	Reject

No: no information or insufficient information

Yes: sufficient information

n/a: not applicable

### The clinically relevant characteristics of the recommended observation methods

Table 4 presents the main characteristics of the two observation methods retained: the BAT-Back and the PaBS.

**Table 4:** Recommended observation methods suitable for utilization in clinical settings

Tool	Behavioral avoidance test (BAT-BACK) [27]	Pain Behaviour Scale [36]
**What does the tool measure?**	Measures observable avoidance behaviors. May be used to plan graded exposure for patients with chronic lumbar pain or as a tool to measure therapeutic success.	Measures observable pain behaviors.
**How is the tool administered?**	The patient must approach the feared stimulus in a standardized environment to induce fear and avoidance reactions 1. Instructions are given to the patient 2. Demonstration of movements (bending forward, lifting a box ~ 8 kg, rotation) by the evaluator 3. Movements are executed by the patient (10 repetitions) 4. Assessment of behavior (according to 3 categories)	The patient performs a standardized sequence of physical performance tests 1. Repeated trunk flexion 2. Repeated sit to stand 3. Timed up and go 4. Loaded reach 5. 50-ft walk
**Observed PB**	Category 1: The movement is carried out as demonstrated by the evaluator. No avoidance or protective behavior. Category 2: The movement is carried out with protective behaviors (bended knees, keep the back straight by lifting or bending, move feet while turning, deep breaths, taking medication before the task, drinking water, seeking support, asking for help). Category 3: The patient avoids making the movement. If less than 10 repetitions, missing repetitions are scored as avoided.	The specific pain behaviors assessed are: - Sighing - Breath-holding, - Grimacing - Guarding - Rubbing - Antalgic gait
**Scoring and interpretation of the observation method**	Each repetition is scored as follows: Category 1 = 0 point Category 2 = 1 point Category 3 = 2 points Thus, a score of 0 means that the patient avoided no movement or did not engage in a protective movement, and a score of 60 means that the patient avoided all movements.	For each task, the intensity and severity of PB are rated as below: a) Intensity Presence or absence of each PB b) Severity For each task, determination of PB severity with a 4-point scale: 0. None 1. Mild 2. Moderate 3. Severe Finally, a total severity score (0–15) is obtained with the sum of the 5-task PB severity score.
**Clinical benefits**	• Easy to administer and interpret • Short (approx. 5 min) • Requires little to no material	• Easy to administer • Short (10–15 min) and assess physical performance at the same time • Requires little to no material • Also informs on physical performance
**Validity and Reliability**	• The BAT-Back is a reliable and valid measure of pain avoidance behavior Inter-rater reliability: good to excellent • Internal consistency: excellent • Convergent validity and divergent validity were determined • Cross-cultural validity (Turkish) [40]	• The PaBS is a reliable and valid measure to assess the presence and severity of PB. • Inter-rater reliability: excellent • Intra-rater reliability: excellent • Agreement for each PB in each task between 95 and 100% • Perfect consistency for the absence/presence of PB • Acceptable construct validity
**Target population**	People with chronic low back pain. (CLBP) Participants in the validity study were between 18 and 65 years old.	People with chronic low back pain Participants in this study were between 21 and 65 years old.

### Listing of the observable PB identified from the literature

For the second objective, 3360 relevant articles were found in the various databases consulted. Two more articles were included after an exploratory research. After the removal of duplicates (1488 excluded), title/abstract screening (1694 excluded with 112 abstracts not available), we obtained a pool of 180 articles. From this pool, 28 articles were not available, 45 were excluded for failure to meet inclusion criteria, which left 107 articles for review. See Fig. 1 for the flow chart diagram.

Based on the extracted data from the 107 studies, 21 different groups of PB were identified. We grouped together similar PB and we classified these groups into 7 categories: (1) verbal and non-verbal communication, (2) sounds, (3) posture and movements, (4) inconsistent findings during clinical examination, (5) physical activities, (6) social and occupational activities, and (7) inappropriate use of. The complete listing and categories are presented in Table 5**.**

**Table 5:** Listing of observable pain behaviors found in the systematic search and review

	Does the patient exhibit this PB? If yes, with the integration of the patient’s context, beliefs, the frequency of this PB, etc., the clinician can interpret if the PB is adaptive or not and interferes with the rehabilitation process
**Verbal and nonverbal communication** *Pain-related behaviors that can be verbalized or executed by the patient to communicate with the therapist about his/her pain*	
• The patient stays focused on pain communication (e.g., always refers to his/her pain during conversation) [28, 29, 35, 41–56]	
• The patient stays focused on disability or impairments despite clinical improvements [43, 52, 54, 57–60]	
• The patient verbalizes hesitation or questions about his/her capacity to perform feasible tasks [42, 54, 55, 58, 61]	
• The patient asks for help for tasks he/she can perform independently (alone) [27, 44–46, 49, 55, 60, 62–65]	
Touching/rubbing the painful area after task accomplishment [11, 18, 28, 29, 31–36, 39, 42, 45, 51–56, 58, 61, 66–111]	
**Sounds** *Pain-related behaviors that can be heard by the therapist when the patient performs tasks or activities*	
• Groaning, Moaning, Whining, Whimpering, Crying, Screaming [11, 18, 27–30, 34, 35, 37, 39, 42, 44–47, 49–51, 53–59, 61–63, 67, 68, 70–74, 88, 97, 103–105, 107, 109, 111–114]	
Sighing, Holding their breath, Taking a deep breath [11, 18, 27–36, 39, 42, 44, 50, 53–57, 61, 66, 68, 69, 71–74, 76–82, 85–87, 89–94, 96, 97, 100–103, 106, 107, 109–111, 115, 116]	
**Posture and movements** *Pain-related behaviors that can be seen by the therapist when the patient moves or remains in a static position*	
• Overcautious/overprotective during movements *○ Self-limiting range of motion* *○ Stiff or rigid movements* *○ Abnormally slow movements* [11, 18, 26, 28, 29, 31–38, 42, 44–46, 49, 52, 53, 55, 57–59, 61–63, 66–69, 71, 72, 74–82, 85–87, 89, 90, 92–104, 106, 107, 109–111, 116–122]	
• Strategies to minimize the threat and/or the load on the painful area during movement *○ Avoids or minimizes lifting, bending* *○ Bending knees, kneeling, keeping the back straight* *○ Moving the feet while rotating* *○ Imbalance on the distribution of body weight* [11, 18, 27, 28, 30, 32, 33, 37, 42, 45, 50, 52–59, 61–63, 66, 67, 69–71, 74–89, 92–103, 105–110, 119, 120, 123, 124]	
• Keeps distorted gait despite clinical improvements *○ Limps* *○ Drag one’s leg* [11, 29, 33–36, 39, 41, 45, 46, 48, 49, 52, 54–56, 58, 62–64, 73, 74, 83, 84, 88, 91, 105, 113, 115]	
• Delays activity execution *○ Drinks water between the order and the performance of a requested task/movement* *○ Latency to initiate a requested task/movement* *○ Misses therapy sessions if not reminded* [27, 55, 58, 117, 120]	
• Excessive rest [34, 35, 43, 44, 51, 54, 55, 60, 62, 63, 70, 74, 84, 88, 105, 118, 119, 124–128]	
**Inconsistent findings during clinical examination** *Pain-related behaviors that can be provoked during the clinical examination*	
• Discrepancies between: *○ clinical findings and observed functional capacity or incapacity (dressing, …)* *○ the demonstrated range of motion during clinical examination and during distraction tasks* [42, 129, 135]	
• Overreaction during examination [42, 129, 135]	
**Physical activities** *Pain-related behaviors that can be mentioned by the patient while talking about physical activities or performing tasks*	
• Avoid or minimize: *○ leisure activities* *○ housework* *○ sports* *○ sexual intercourse* [11, 33, 37, 43, 50, 51, 54–56, 60, 65, 67, 84, 125, 128, 130, 136]	
• Undertakes nothing outside therapy time despite therapist’s encouragement [55]	
**Social and occupational activities** *Pain-related behaviors that can be mentioned by the patient while talking about social or occupational activities*	
• Avoids or minimizes spending time with people [50, 51, 55, 56, 62, 63, 74, 125]	
• Repeated work absences [51, 56]	
**Inappropriate use of** *Pain-related behaviors that can be mentioned by the patient while talking about or seen by the therapist*	
• Medication (prescribed or not) [27, 29, 45, 46, 51, 54–56, 58, 60, 67, 88, 105, 118, 119, 126, 127]	
• Healthcare system *○ Asking for further specialized medical treatment* [51, 55]	
• Non-prescribed equipment *○ TENS* *○ Cane or crutch* *○ Brace* [27, 29, 37, 39, 44, 46, 49, 52, 54, 55, 58, 62, 63, 67, 70, 86–88, 105, 119, 127]	

## Discussion

To our knowledge, this study is the first exhaustive and comprehensive review to critically appraise observation methods to assess PB considering clinical and psychometric criteria, identify, and categorize PB described in the literature that patients can exhibit. Concerning the assessment tools, our review shows that observation methods easily applied in clinical practice are scarce. We extracted from the literature, a large spectrum of possible PB that may be observed, from subtle behaviors (e.g. drink water to delay task) to more obvious (e.g. avoidance of the painful task). Clinicians may benefit from awareness of the different PB clinical presentations to detect maladaptive behaviors in people with musculoskeletal pain, which often suggest the presence of cognitive-emotional factors that may interfere with the rehabilitation process.

The clinical criteria of our triage process allowed us to select 3 observation methods, but the psychometric assessment suggests that the TIPPA presented low methodological quality. Thus, we recommend the use of the PaBS or the BAT-Back in clinical practice since both of these scored well during the psychometric assessment. However, these two observation methods are only validated for people with chronic low back pain which limits the objective assessment of maladaptive PB of other musculoskeletal conditions. It is worth noting that these two observation methods present some differences and limitations. The first main difference involves instrument scoring. The BAT-Back proposes a score of avoidance, and more precisely physical avoidance (protective PBs) [27], as opposed to the PaBS, which proposes a severity score within a range of protective or communicative PBs [36]. As a result, the PaBS allowed clinicians to evaluate a larger diversity of PB.

Clinicians must use caution when evaluating communicative PBs, as they are not always related to pain severity and pain-related disability [28]. Moreover, observers give more weight to communicative PBs than protective behaviors [28]. This overinterpretation and its consequences on clinicians’ attitudes towards the patient may lead to reinforce these communicative PBs [28]. Thus, despite the lack of diversity in the PBs it assesses, the BAT-Back’s focus on protective PBs may avoid this reinforcement, while providing information about pain severity and pain-related disability [28]. The difference in the type of PBs assessed does not seem to be a limitation.

The BAT-Back scoring can be confusing as it is based on a sequence of 3 movements. If a patient stops the sequence during the first movement, the remaining two are not performed, but scored as avoided movements, which can lead to an overestimation of avoidance [40]. Furthermore, as the first movement is bending forward, its scoring can be influenced (biased) by physical consequences of underactivity such as stiffness, shortness of muscles, among many other factors [40]. For example, if the patient bends to the knees or keeps his back straight, the BAT-Back considers that the patient engages in safety behaviors. Physical limitations, such as less flexible hamstrings may lead to an overestimation of patient avoidance. The score based on a sequence and the rating that can be influenced by physical or cognitive consequences of the patient’s life are the main limitations of the BAT-Back. Another limitation of the BAT-Back is the tasks that are performed. Even if the 3 movements of the BAT-Back are known to be fearful tasks for patients with low back pain, it is also well known that a patient can avoid certain tasks, but can perform others without avoidance [66]. The PaBS uses tasks from the physical performance assessment to evaluate PBs. With this strategy, the PaBS increases the number of tasks that are performed. However, all the tasks performed for the PaBS are in a sagittal plan whereas the BAT-Back uses movements in the sagittal and horizontal plans.

Our results also highlights the ubiquitous of the avoidance behaviors reported in the literature, as the types of most PB found in the literature were either protective or communicative. This discrepancy could be explained by the fact that the Fear-Avoidance Model was conceptually proposed in 2000 [6], whereas the Endurance-Avoidance Model was conceptually proposed 10 years later [9]; not surprisingly, much more literature is based on the Fear-Avoidance Model. Another reason relates to the behaviors themselves. Contrary to the avoidance response, pattern that is characterized by pain-related fear, catastrophizing, and behavioral avoidance [6], the endurance response pattern is characterized by thought suppression, anxiety/depression, and task persistence (endurance behaviors) [7]. Thus, even if avoidance behaviors are subtle, they still remain observable [67]. On the other hand, as “endurers” carry out the task to the end despite a significant increase in pain [131], endurance behaviors seem less “observable” and could be better captured by a questionnaire. If a clinician suspects that his or her patient presents endurance behaviors when performing a task, it would seem more appropriate to use a questionnaire such as the Avoidance Endurance Questionnaire to assess the patient’s PB [125, 132–134].

The assessment of behavioral components is an integral part of a biopsychosocial approach. However, clinicians can feel uncomfortable in the assessment of psychosocial factors [26] and want the support of simple screening tools [29]. Also, because PB are dynamic (adapted in the short term and can turn into maladaptive behavior), it is essential to have the possibility of a rapid screening. With a screening perspective, we summarized the different observable PB found in the literature. Yet, when available, clinicians must also objectively document these with a proper tool. In this case, the PaBS or the BAT-Back can be used.

Our systematic search and review present some limitations. The first one concerns the clinical criteria used to select the observation methods. As no specific tools were available, we had to create our own grid based on data from the literature on the clinical integration of outcome measures in rehabilitation. The second main limitation concerns the clinical assessment tool (CAT) developed by Brink and Louw. Although cited in several studies (*n* = 40), this CAT is not validated for this type of analysis.

## Conclusion

This is the first review to identify and critically appraise observation methods to assess pain behaviors in patients with musculoskeletal pain in clinical setting. The critical appraisal process allowed us to recommend two observation methods that are rapid to complete, with few equipment, and using tasks perceived as threatening by patients. These methods are the PaBS and the BAT-Back. However, these two tools are only validated for people with chronic low back pain. In order to help clinicians in the detection of possible maladaptive PBs in patients with various musculoskeletal conditions, we extracted the different PBs present in the literature and that patients can exhibit. This extraction allowed us to propose 7 categories of PBs. With that, clinicians can perform a screening of PBs, but not an objective assessment. Also, this review shows the ubiquitous of the avoidance behaviors in the literature. Thus, clinicians may use a questionnaire like the Avoidance Endurance Questionnaire to perform a global evaluation of behaviors that can be part of the two models of transition to chronicity.

## Data Availability

The datasets used and/or analysed during the current study are available from the corresponding author upon reasonable request.

## References

[1] Tousignant-Laflamme Y, Martel MO, Joshi AB, Cook CE (2017). Rehabilitation management of low back pain–it’s time to pull it all together!. J Pain Res.

[2] Foster NE, DeLitto A (2011). Embedding psychosocial perspectives within clinical management of low back pain: integration of informed management principles into physical therapy practice - challenges and opportunities. Phys Ther.

[3] Wijma AJ, van Wilgen CP, Meeus M, Nijs J (2016). Clinical biopsychosocial physiotherapy assessment of patients with chronic pain: the first step in pain neuroscience education. Physiother Theory Pract.

[4] Meints SM, Edwards RR (2018). Evaluating psychosocial contributions to chronic pain outcomes. Prog Neuro-Psychopharmacol Biol Psychiatry.

[5] Lin I, Wiles L, Waller R, Goucke R, Nagree Y, Gibberd M, et al. What does best practice care for musculoskeletal pain look like? Eleven consistent recommendations from high-quality clinical practice guidelines: systematic review. Br J Sports Med. 2019:bjsports-2018-099878 10.1136/BJSPORTS-2018-099878. 10.1136/bjsports-2018-099878 30826805

[6] Vlaeyen JW, Linton SJ (2000). Fear-avoidance and its consequences in chronic musculoskeletal pain: a state of the art. Pain.

[7] Hasenbring MI, Chehadi O, Titze C, Kreddig N (2014). Fear and anxiety in the transition from acute to chronic pain: there is evidence for endurance besides avoidance. Pain Manag.

[8] Fehrmann E, Tuechler K, Kienbacher T, Mair P, Spreitzer J, Fischer L, Kollmitzer J, Ebenbichler G (2017). Comparisons in muscle function and training rehabilitation outcomes between avoidance-endurance model subgroups. Clin J Pain.

[9] Hasenbring MI, Verbunt JA (2010). Fear-avoidance and endurance-related responses to pain: new models of behavior and their consequences for clinical practice. Clin J Pain.

[10] Wertli MM, Rasmussen-Barr E, Held U, Weiser S, Bachmann LM, Brunner F (2014). Fear-avoidance beliefs—a moderator of treatment efficacy in patients with low back pain: a systematic review. Spine J.

[11] Martel MO, Sullivan MJL. Pain behavior: Unitary or multidimensional phenomenon? Soc Interpers Dyn Pain We Dont Suff Alone. 2018:79–99 10.1007/978-3-319-78340-6_5.

[12] Fordyce WE, Shelton JL, Dundore DE (1982). The modification of avoidance learning pain behaviors. J Behav Med.

[13] Vlaeyen JWS, Linton SJ (2012). Fear-avoidance model of chronic musculoskeletal pain: 12 years on. Pain.

[14] Svege I, Fernandes L, Nordsletten L, Holm I, Risberg MA (2016). Long-term effect of exercise therapy and patient education on impairments and activity limitations in people with hip osteoarthritis: secondary outcome analysis of a randomized clinical trial. Phys Ther.

[15] Friedrich M, Gittler G, Arendasy M, Friedrich KM (2005). Long-term effect of a combined exercise and motivational program on the level of disability of patients with chronic low back pain. Spine.

[16] Li Q, Loke AY (2014). A systematic review of spousal couple-based intervention studies for couples coping with cancer: direction for the development of interventions. Psychooncology.

[17] O’Sullivan PB, Caneiro JP, O’Keeffe M, Smith A, Dankaerts W, Fersum K, O’Sullivan K (2018). Cognitive functional therapy: An integrated behavioral approach for the targeted Management of Disabling low Back Pain. Phys Ther.

[18] Prkachin KM, Hughes E, Schultz I, Joy P, Hunt D (2002). Real-time assessment of pain behavior during clinical assessment of low back pain patients. Pain.

[19] Darlow B, Fullen BM, Dean S, Hurley DA, Baxter GD, Dowell A (2012). The association between health care professional attitudes and beliefs and the attitudes and beliefs, clinical management, and outcomes of patients with low back pain: a systematic review. Eur J Pain.

[20] Grant MJ, Booth A (2009). A typology of reviews: an analysis of 14 review types and associated methodologies. Health Inf Libr J.

[21] Al-Muqiren TN, Al-Eisa ES, Alghadir AH, Anwer S (2017). Implementation and use of standardized outcome measures by physical therapists in Saudi Arabia: barriers, facilitators and perceptions. BMC Health Serv Res.

[22] Duncan EA, Murray J (2012). The barriers and facilitators to routine outcome measurement by allied health professionals in practice: a systematic review. BMC Health Serv Res.

[23] Brink Y, Louw QA (2012). Clinical instruments: reliability and validity critical appraisal. J Eval Clin Pract.

[24] Silva AG, Simões P, Queirós A, Rodrigues M, Rocha NP (2020). Mobile apps to quantify aspects of physical activity: a systematic review on its reliability and validity. J Med Syst.

[25] Wu H-D, Liu W, Wong M-S (2020). Reliability and validity of lateral curvature assessments using clinical ultrasound for the patients with scoliosis: a systematic review. Eur Spine J Off Publ Eur Spine Soc Eur Spinal Deform Soc Eur Sect Cerv Spine Res Soc.

[26] Holopainen R, Simpson P, Piirainen A, Karppinen J, Schütze R, Smith A, O'Sullivan P, Kent P (2020). Physiotherapists’ perceptions of learning and implementing a biopsychosocial intervention to treat musculoskeletal pain conditions: a systematic review and metasynthesis of qualitative studies. Pain.

[27] Holzapfel S, Riecke J, Rief W, Schneider J, Glombiewski JA (2016). Development and validation of the behavioral avoidance test-Back pain (BAT-Back) for patients with chronic low Back pain. Clin J Pain.

[28] Sullivan MJL, Thibault P, Savard A, Catchlove R, Kozey J, Stanish WD (2006). The influence of communication goals and physical demands on different dimensions of pain behavior. Pain.

[29] Driver C, Lovell GP, Oprescu F (2021). Psychosocial strategies for physiotherapy: a qualitative examination of physiotherapists’ reported training preferences. Nurs Health Sci.

[30] Aung M, Bianchi-Berthouze N, Watson P, Williams A (2014). Automatic Recognition of Fear-Avoidance behavior in Chronic Pain Physical Rehabilitation.

[31] Cinciripini PM, Floreen A (1983). An assessment of chronic pain behavior in a structured interview. J Psychosom Res.

[32] Andersen TE, Ravn SL, Manniche C, O’Neill S (2018). The impact of attachment insecurity on pain and pain behaviors in experimental pain. J Psychosom Res.

[33] Keefe FJ, Wilkins RH, Cook WA (1984). Direct observation of pain behavior in low back pain patients during physical examination. Pain.

[34] Burns JW, Quartana P, Gilliam W, Gray E, Matsuura J, Nappi C, Wolfe B, Lofland K (2008). Effects of anger suppression on pain severity and pain behaviors among chronic pain patients: evaluation of an ironic process model. Health Psychol.

[35] Keefe FJ, Hill RW (1985). An objective approach to quantifying pain behavior and gait patterns in low back pain patients. Pain 03043959.

[36] Alamam DM, Leaver A, Moloney N, Alsobayel HI, Alashaikh G, MacKey MG (2019). Pain behaviour scale (PaBS): An exploratory study of reliability and construct validity in a chronic low back pain population. Pain Res Manag.

[37] Missaghi-Wedefalk M, Lindh M, Schön-Ohlsson C, WilléN C (2012). Further methodological development of the test instrument for profile of physical ability (TIPPA) designed for patients with long-term musculoskeletal pain. Adv Physiother.

[38] Koho P, Aho S, Watson P, Hurri H (2001). Assessment of chronic pain behaviour: reliability of the method and its relationship with perceived disability, physical impairment and function. J Rehabil Med.

[39] Moores LL, Watson PJ (2004). The development of a measurement tool for the assessment of pain behaviour in real time. Physiotherapy.

[40] Küçükakkaş O, Karaman ÇA (2020). Cross-cultural adaptation and validation of the behavioral avoidance test-Back pain (BAT-Back) to the Turkish language. J Orthop Sci.

[41] Thieme K, Spies C, Sinha P, Turk DC, Flor H (2005). Predictors of pain behaviors in fibromyalgia syndrome. Arthritis Care Res.

[42] Watson PJ, Poulter ME (1997). The development of a functional task-oriented measure of pain behaviour in chronic low back pain patients. J Back Musculoskelet Rehabil.

[43] Mohammadi S, Chambers CT, Rosen NO (2018). Expression of pain behaviors and perceived partner responses in individuals with chronic pain. Clin J Pain.

[44] Meyer K, Klipstein A, Oesch P, Jansen B, Kool J, Niedermann K (2016). Development and validation of a pain behavior assessment in patients with chronic low Back pain. J Occup Rehabil.

[45] Anciano D (1986). The pain behaviour checklist: factor analysis and validation. Br J Clin Psychol.

[46] Cook KF, Keefe F, Jensen MP, Roddey TS, Callahan LF, Revicki D, Bamer AM, Kim J, Chung H, Salem R, Amtmann D (2013). Development and validation of a new self-report measure of pain behaviors. Pain.

[47] Revicki DA, Chen W-H, Harnam N, Cook KF, Amtmann D, Callahan LF, Jensen MP, Keefe FJ (2009). Development and psychometric analysis of the PROMIS pain behavior item bank. Pain.

[48] Kerns RD, Haythornthwaite J, Rosenberg R, Southwick S, Giller EL, Jacob MC (1991). The pain behavior check list (PBCL): factor structure and psychometric properties. J Behav Med.

[49] Leung SM, Chung J (2008). Beliefs about appropriate pain behaviour: gender differences between health care professionals and non-health care professionals in Hong Kong. J Clin Nurs.

[50] Mohammadi S, Dehghani M, Sanderman R, Hagedoorn M (2017). The role of pain behaviour and family caregiver responses in the link between pain catastrophising and pain intensity: a moderated mediation model. Psychol Health.

[51] Osman A, Barrios FX, Kopper B, Osman JR, Grittmann L, Troutman JA, Panak WJ (1995). The pain behavior check list (PBCL): psychometric properties in a college sample. J Clin Psychol.

[52] Philips HC (1987). Avoidance behaviour and its role in sustaining chronic pain. Behav Res Ther.

[53] Radnitz CL, Appelbaum KA, Blanchard EB, Elliott L, Andrasik F (1988). The effect of self-regulatory treatment on pain behavior in chronic headache. Behav Res Ther.

[54] Romano JM, Turner JA, Friedman LS, Bulcroft RA, Jensen MP, Hops H, Wright SF (1992). Sequential analysis of chronic pain behaviors and spouse responses. J Consult Clin Psychol.

[55] Sullivan MJL, Davidson N, Garfinkel B, Siriapaipant N, Scott W (2009). Perceived injustice is associated with heightened pain behavior and disability in individuals with whiplash injuries. Psychol Inj Law.

[56] Turk DC, Wack JT, Kerns RD (1985). An empirical examination of the “pain-behavior” construct. J Behav Med.

[57] Vlaeyen JW, Van Eek H, Groenman NH, Schuerman JA (1987). Dimensions and components of observed chronic pain behavior. Pain.

[58] Philips HC, Jahanshahi M (1986). The components of pain behaviour report. Behav Res Ther.

[59] Ahern DK, Hannon DJ, Goreczny AJ, Follick MJ, Parziale JR (1990). Correlation of chronic low-back pain behavior and muscle function examination of the flexion-relaxation response. Spine.

[60] Follick MJ, Ahern DK, Aberger EW (1985). Development of an audiovisual taxonomy of pain behavior: reliability and discriminant validity. Health Psychol.

[61] Kleinke CL, Spangler AS (1988). Psychometric analysis of the audiovisual taxonomy for assessing pain behavior in chronic back-pain patients. J Behav Med.

[62] Singh AN (2012). Pain behaviours and psychiatric complications in pain syndrome. Int Med J.

[63] Paulsen JS, Altmaier EM (1995). The effects of perceived versus enacted social support on the discriminative cue function of spouses for pain behaviors. Pain.

[64] Crins MHP, Roorda LD, Smits N, de Vet HCW, Westhovens R, Cella D, Cook KF, Revicki D, van Leeuwen J, Boers M, Dekker J, Terwee CB (2016). Calibration of the Dutch-Flemish PROMIS pain behavior item bank in patients with chronic pain. Eur J Pain.

[65] Schuller W, Terwee CB, Klausch T, Roorda LD, Rohrich DC, Ostelo RW, Terluin B, de Vet HCW (2019). Psychometric properties of the Dutch-Flemish patient-reported outcomes measurement information system pain behavior item bank in patients with musculoskeletal complaints. J Pain.

[66] Keefe FJ, Dunsmoret J (1992). Pain behavior concepts and controversies. APS J.

[67] Volders S, Boddez Y, De Peuter S, Meulders A, Vlaeyen JWS (2015). Avoidance behavior in chronic pain research: a cold case revisited. Behav Res Ther.

[68] Shen MJ, Redd WH, Winkel G, Badr H (2014). Associations among pain, pain attitudes, and pain behaviors in patients with metastatic breast cancer. J Behav Med.

[69] Spada MM, Gay H, Nikčevic AV, Fernie BA, Caselli G (2016). Meta-cognitive beliefs about worry and pain catastrophising as mediators between neuroticism and pain behaviour. Clin Psychol.

[70] Prkachin KM, Schultz IZ, Hughes E (2007). Pain behavior and the development of pain-related disability: the importance of guarding. Clin J Pain.

[71] Martel MO, Thibault P, Sullivan MJL (2010). The persistence of pain behaviors in patients with chronic back pain is independent of pain and psychological factors. Pain.

[72] Löfvander MB, Furhoff A-K (2002). Pain behaviour in young immigrants having chronic pain: An exploratory study in primary care. Eur J Pain.

[73] Labus JS, Keefe FJ, Jensen MP (2003). Self-reports of pain intensity and direct observations of pain behavior: when are they correlated?. Pain.

[74] Gauthier N, Thibault P, Sullivan MJL (2011). Catastrophizers with chronic pain display more pain behaviour when in a relationship with a low catastrophizing spouse. Pain Res Manag.

[75] Burns JW, Gerhart J, Post KM, Smith DA, Porter LS, Buvanendran A, Fras AM, Keefe FJ (2018). Spouse criticism/hostility toward partners with chronic pain: the role of spouse attributions for patient control over pain behaviors. J Pain.

[76] Vlaeyen JW, Pernot DF, Kole-Snijders AM, Schuerman JA, Van Eek H, Groenman NH (1990). Assessment of the components of observed chronic pain behavior: the checklist for interpersonal pain behavior (CHIP). Pain.

[77] Ahles TA, Coombs DW, Jensen L, Stukel T, Maurer LH, Keefe FJ (1990). Development of a behavioral observation technique for the assessment of pain behaviors in cancer patients. Behav Ther.

[78] Anderson KO, Bradley LA, Turner RA, Agudelo CA, Pisko EJ, Salley AN, Fletcher KE (1992). Observation of pain behavior in rheumatoid arthritis patients during physical examination. Relationship to disease activity and psychological variables. Arthritis Care Res Off J Arthritis Health Prof Assoc.

[79] Anderson KO, Keefe FJ, Bradley LA, McDaniel LK, Young LD, Turner RA (1988). Prediction of pain behavior and functional status of rheumatoid arthritis patients using medical status and psychological variables. Pain 03043959.

[80] Anderson KO, Bradley LA, Turner RA, Agudelo CA, Pisko EJ (1994). Pain behavior of rheumatoid arthritis patients enrolled in experimental drug trials. Arthritis Care Res.

[81] Bradley LA, Turner RA, Young LD, Agudelo CA, Anderson KO, McDaniel LK (1985). Effects of cognitive-behavioral therapy on pain behavior of rheumatoid arthritis (RA) patients: preliminary outcomes. Scand J Behav Ther.

[82] Buckelew SP, Parker JC, Keefe FJ, Deuser WE, Crews TM, Conway R (1994). Self-efficacy and pain behavior among subjects with fibromyalgia. Pain 03043959.

[83] Burns JW, Quartana P, Bruehl S (2011). Anger suppression and subsequent pain behaviors among chronic low back pain patients: moderating effects of anger regulation style. Ann Behav Med.

[84] Burns JW, Post KM, Smith DA, Porter LS, Buvanendran A, Fras AM, Keefe FJ (2019). Spouse and patient beliefs and perceptions about chronic pain: effects on couple interactions and patient pain behavior. J Pain.

[85] Cinciripini PM (1983). Stimulus control and chronic pain behavior. A study of low back and head/neck/face pain patients. Behav Modif.

[86] Clark SM, Leonard MT, Cano A, Pester B. Beyond operant theory of observer reinforcement of pain behavior. Soc Interpers Dyn Pain We Dont Suff Alone. 2018:273–93 10.1007/978-3-319-78340-6_13.

[87] Connally GH, Sanders SH (1991). Predicting low back pain patients’ response to lumbar sympathetic nerve blocks and interdisciplinary rehabilitation: the role of pretreatment overt pain behavior and cognitive coping strategies. Pain 03043959.

[88] Cook KF, Roddey TS, Bamer AM, Amtmann D, Keefe FJ (2013). Validity of an observation method for assessing pain behavior in individuals with multiple sclerosis. J Pain Symptom Manag.

[89] Dekker J, Winckers M, Tola P, Aufdemkampe G (1993). Categories of pain behaviour in osteoarthritis patients. Physiother Theory Pract.

[90] Feuerstein M, Greenwald M, Gamache MP, Papciak AS, Cook EW (1985). The pain behavior scale: modification and validation for outpatient use. J Psychopathol Behav Assess.

[91] Gil KM, Keefe FJ, Crisson JE, Van Dalfsen PJ (1987). Social support and pain behavior. Pain 03043959.

[92] Gil KM, Phillips G, Edens J, Martin NJ, Abrams M (1994). Observation of pain behaviors during episodes of sickle cell disease pain. Clin J Pain.

[93] Harper P (2006). No pain, no gain: pain behaviour in the armed forces. Br J Nurs Mark Allen Publ.

[94] Keefe FJ, Wilkins RH, Cook WA, Crisson JE, Muhlbaier LH (1986). Depression, pain, and pain behavior. J Consult Clin Psychol.

[95] Keefe FJ, Dolan E (1986). Pain behavior and pain coping strategies in low back pain and myofascial pain dysfunction syndrome patients. Pain.

[96] Keefe FJ (2000). Pain behavior observation: current status and future directions. Curr Rev Pain.

[97] Keefe FJ, Lefebvre JC, Egert JR, Affleck G, Sullivan MJ, Caldwell DS (2000). The relationship of gender to pain, pain behavior, and disability in osteoarthritis patients: the role of catastrophizing. Pain 03043959.

[98] Keefe FJ, Bradley LA, Crisson JE (1990). Behavioral assessment of low back pain: identification of pain behavior subgroups. Pain 03043959.

[99] Martel M-O, Trost Z, Sullivan MJ (2012). The expression of pain behaviors in high catastrophizers: the influence of automatic and controlled processes. J Pain Off J Am Pain Soc.

[100] Martel MO, Thibault P, Sullivan MJL (2011). Judgments about pain intensity and pain genuineness: the role of pain behavior and judgmental heuristics. J Pain.

[101] Martel MO, Wideman TH, Sullivan MJL (2012). Patients who display protective pain behaviors are viewed as less likable, less dependable, and less likely to return to work. Pain.

[102] McCahon S, Strong J, Sharry R, Cramond T (2005). Self-report and pain behavior among patients with chronic pain. Clin J Pain.

[103] McDaniel LK, Anderson KO, Bradley LA, Young LD, Turner RA, Agudelo CA (1986). Development of an observation method for assessing pain behavior in rheumatoid arthritis patients. Pain.

[104] Multon KD, Parker JC, Smarr KL, Stucky RC, Petroski G, Hewett JE, Wright GE, Rhee SH, Walker SE (2001). Effects of stress management on pain behavior in rheumatoid arthritis. Arthritis Rheum Arthritis Care Res.

[105] Prkachin KM, Schultz I, Berkowitz J, Hughes E, Hunt D (2002). Assessing pain behaviour of low-back pain patients in real time: concurrent validity and examiner sensitivity. Behav Res Ther.

[106] Puntillo KA, Morris AB, Thompson CL, Stanik-Hutt J, White CA, Wild LR (2004). Pain behaviors observed during six common procedures: results from thunder project II. Crit Care Med.

[107] Richards JS, Nepomuceno C, Riles M, Suer Z (1982). Assessing pain behavior: the UAB Pain Behavior Scale. Pain 03043959.

[108] Romano JM, Syrjala KL, Levy RL, Turner JA, Evans P, Keefe FJ (1988). Overt pain behaviors: relationship to patient functioning and treatment outcome. Behav Ther.

[109] Sullivan MJL, Adams H, Sullivan ME (2004). Communicative dimensions of pain catastrophizing: social cueing effects on pain behaviour and coping. Pain 03043959.

[110] Vigil JM, Coulombe P (2011). Biological sex and social setting affects pain intensity and observational coding of other people’s pain behaviors. Pain.

[111] Waddell G, Richardson J (1992). Observation of overt pain behaviour by physicians during routine clinical examination of patients with low back pain. J Psychosom Res.

[112] Waters SJ, Riordan PA, Keefe FJ, Lefebvre JC (2008). Pain behavior in rheumatoid arthritis patients: identification of pain behavior subgroups. J Pain Symptom Manag.

[113] Sullivan MJL, Tripp DA, Santor D (2000). Gender differences in pain and pain behavior: the role of catastrophizing. Cogn Ther Res.

[114] Appelbaum KA, Radnitz CL, Blanchard EB, Prins A (1988). The pain behavior questionnaire (PBQ): a global report of pain behavior in chronic headache. Headache J Head Face Pain.

[115] Badr H, Milbury K (2011). Associations between depression, pain behaviors, and partner responses to pain in metastatic breast cancer. Pain.

[116] Cautela JR (1977). The use of covert conditioning in modifying pain behavior. J Behav Ther Exp Psychiatry.

[117] Carriere JS, Martel M-O, Kao M-C, Sullivan MJ, Darnall BD (2017). Pain behavior mediates the relationship between perceived injustice and opioid prescription for chronic pain: a collaborative health outcomes information registry study. J Pain Res.

[118] Bradley LA, Young LD, Anderson KO, Turner RA, Agudelo CA, McDaniel LK (1987). Effects of psychological therapy on pain behavior of rheumatoid arthritis patients. Treatment outcome and six-month followup. Arthritis Rheum.

[119] Ashton-James CE, Richardson DC, De Williams ACC, Bianchi-Berthouze N, Dekker PH (2014). Impact of pain behaviors on evaluations of warmth and competence. Pain.

[120] Keefe FJ, Smith S (2002). The assessment of pain behavior: implications for applied psychophysiology and future research directions. Appl Psychophysiol Biofeedback.

[121] Ohlund C, Lindström I, Areskoug B, Eek C, Peterson LE, Nachemson A (1994). Pain behavior in industrial subacute low back pain. Part I. Reliability: concurrent and predictive validity of pain behavior assessments. Pain 03043959.

[122] Prigent E, Amorim M-A, Leconte P, Pradon D (2014). Perceptual weighting of pain behaviours of others, not information integration, varies with expertise. Eur J Pain U K.

[123] Werner P, Al-Hamadi A, Limbrecht-Ecklundt K, Walter S, Traue HC. Head movements and postures as pain behavior. PLoS One. 2018;13(2):e0192767. 10.1371/journal.pone.0192767. 10.1371/journal.pone.0192767 PMC5812618 29444153

[124] Nagarajan M, Nair MR (2010). Importance of fear-avoidance behavior in chronic non-specific low back pain. J Back Musculoskelet Rehabil.

[125] An J, Kim YH, Cho S (2018). Validation of the Korean version of the avoidance endurance behavior questionnaire in patients with chronic pain. Health Qual Life Outcomes.

[126] Roberts L, Little P, Chapman J, Cantrell T, Pickering R, Langridge J (2002). The back home trial: general practitioner-supported leaflets may change back pain behavior. Spine.

[127] Pence LB, Thorn BE, Jensen MP, Romano JM (2008). Examination of perceived spouse responses to patient well and pain behavior in patients with headache. Clin J Pain.

[128] Estlander AM (1989). Determinants of pain behaviour in patients with chronic low back pain. Ann Med.

[129] Krause SJ, Wiener RL, Tait RC. Depression and pain behavior in patients with chronic pain. Clin J Pain. 1994;10(2):122–7. 10.1097/00002508-199406000-00005 8075464

[130] Jensen MP, Ward LC, Thorn BE, Ehde DM, Day MA (2017). Measuring the cognitions, emotions, and motivation associated with avoidance behaviors in the context of pain: preliminary development of the negative Responsivity to pain scales. Clin J Pain.

[131] Luthi F, Vuistiner P, Favre C, Hilfiker R, Léger B (2018). Avoidance, pacing, or persistence in multidisciplinary functional rehabilitation for chronic musculoskeletal pain: An observational study with cross-sectional and longitudinal analyses. PLoS One.

[132] Hasenbring MI, Hallner D, Rusu AC (2009). Fear-avoidance- and endurance-related responses to pain: development and validation of the avoidance-endurance questionnaire (AEQ). Eur J Pain Lond Engl.

[133] Karimi Ghasem Abad S, Akhbari B, Salavati M, Saeedi A, Seydi M, Shakoorianfard MA (2020). Translation, reliability, and validity of the avoidance endurance questionnaire in Iranian subjects with chronic non-specific neck pain. J Fam Med Prim Care.

[134] Ruiz-Párraga G, López-Martínez A, Rusu A, Hasenbring M (2015). Spanish version of the avoidance-endurance questionnaire: factor structure and psychometric properties. Span J Psychol.

[135] Dickens C, Jayson M, Creed F (2002). Psychological correlates of pain behavior in patients with chronic low back pain. Psychosom J Consult Liaison Psychiatry.

[136] Schwartz L, Slater MA, Birchler GR (1994). Interpersonal stress and pain behaviors in patients with chronic pain. J Consult Clin Psychol.

